# 17β-Estradiol-Loaded Exosomes for Targeted Drug Delivery in Osteoporosis: A Comparative Study of Two Loading Methods

**DOI:** 10.34172/apb.2023.072

**Published:** 2023-02-01

**Authors:** Mohammad Sadegh Gholami Farashah, Maryam Javadi, Jafar Soleimani Rad, Seyed Kazem Shakouri, Solmaz Asnaashari, Siavoush Dastmalchi, Sadeneh Nikzad, Leila Roshangar

**Affiliations:** ^1^Physical Medicine and Rehabilitation Research Center, Aging Research Institute, Tabriz University of Medical Sciences, Tabriz, Iran.; ^2^Biotechnology Research Center, Tabriz University of Medical Sciences, Tabriz, Iran.; ^3^Stem Cell Research Center, Tabriz University of Medical Sciences, Tabriz, Iran.; ^4^Department of Anatomical Sciences, Faculty of medicine, Tabriz University of Medical Sciences, Tabriz, Iran.; ^5^Faculty of Pharmacy, Tabriz University of Medical Sciences, Tabriz, Iran.; ^6^Faculty of Pharmacy, Near East University, POBOX:99138, Nicosia, North Cyprus, Mersin 10, Turkey.; ^7^Biology Department, Concordia University, Montreal, Canada.

**Keywords:** Osteoporosis, Exosome, Drug delivery, 17β-estradiol, Bone marrow mesenchymal stem cells

## Abstract

**Purpose:**

Exosomes are natural nanoparticles that participate in intercellular communication through molecular transport. Recently, due to their membrane vesicular structure and surface proteins, exosomes have been used extensively in the research field of drug delivery. Osteoporosis is an inflammation in which the cellular balance of bone tissue is disturbed that reduces bone density and making bone prone to abnormal fractures with small amount of force. Utilizing estrogen is one of the main therapeutic strategies for osteoporosis. Despite the positive effects of estrogen on bone tissue, changes in the natural estrogen levels of the body can cause a number of diseases such as different types of cancer. Therefore, designing a therapeutic system which controls more accurate tissue targeting of estrogen seems to be a rational and promising practical approach.

**Methods:**

In this study, bone marrow mesenchymal stem cells (BMMSCs)-derived exosomes were loaded by estradiol using two different methods of drug loading, namely incubation and sonication methods and then the survival effects of the drug loaded exosomes on BMMSCs was investigated.

**Results:**

Examination of size, shape, and surface factors of exosomes in different states (pure exosomes and drug-loaded exosomes) showed that the round morphology of exosomes was preserved in all conditions. However, the particles size increased significantly when loaded by sonication method. The increased survival of BMMSCs was noted with estradiol-loaded exosomes when compared to the control group.

**Conclusion:**

The results suggest that estradiol-loaded exosomes have potential to be used as nano-drug carriers in the treatment of osteoporosis.

## Introduction

 Bone is the main element of the skeletal system. From a histological point of view, it is composed of two parts: the cell (osteoblasts and osteoclasts) and the extracellular matrix. A well homeostasis balance in bone requires effective communication between osteoblasts and osteoclasts through cytokines, growth factors, and some other mediators. However, for a variety of reasons, the balanced homeostasis can be disturbed leading to elevated osteoclast activity and a higher bone resorption than bone production by osteoblasts. This imbalance in cellular activity and homeostasis causes a pathological state in bone tissue called osteoporosis.^1^

 The main feature of osteoporosis is decreased density and quality of bone structure that generally causes higher bone fragility.^2^ Various internal and external factors accelerate bone loss process and make bones more prone to fractures. Internal factors are passing highest bone density in youth, hormonal and genetic disorders, vascular and biochemical conditions. On the other hand, physical activity, nutrition, various diseases, consumption of drugs are examples of external factors.^3^ Osteoporosis can be regarded as a risk factor for bone fractures. The incidence of fractures is increased due to the growth of osteoporosis and osteoporosis is becoming more frequent especially due to the growth of the global aging population of both genders. These higher rates of fractures and osteoporosis, especially in elderly population, have caused augmented physical and psychological problems that impose high mortality and heavy costs on the health systems.^2^

 By itself, the aging process is considered as a chronic inflammatory state called “inflamm-ageing”. Many cells, such as macrophages and highly active inflammatory factors participate in this inflammatory process. Not only it causes osteoporosis and consequent bone fractures, but also in the case of possible fractures, it disrupts the repair of the fractured area.^4^

 One of the main drugs to treat osteoporosis is estrogen. It is a key regulating hormone of bone density and the balance between bone formation and absorption. It causes the balance by increasing the proliferation of osteoblasts and decreasing the activity and proliferation of osteoclasts. Estrogen and its derivatives are highly effective factors in homeostasis as well as bone tissue remodeling.^5^ It has three main natural structures, including estrone (E1), 17β-estradiol (E2), and estriol (E3), among which, 17β-estradiol is the main, most stable, and abundant type.^6,7^ Estrogen acts by affecting two types of intracellular receptors: estrogen receptor-alpha (ER-α) and estrogen receptor-beta (ER-β), which are present in different cells of bone tissue.^5,8^ Despite the positive effects of estrogen on bone tissue, its use has a number of limitations of which the most important is its effect on other tissues. Those tissues strongly affected by estrogen are uterus, breasts,^1^ larynx, cardiovascular system and prostate.^9^ Also, long–term oral and intravenous administration of estrogen in high doses can cause serious side effects and even adverse effects.^1^

 Exosomes are small vesicular particles secreted by most of the cells. These particles are about 30 to 150 nanometers in size and have a round or spherical-shaped lipid bilayer structure. Exosomes have specific receptors on their surface making them very efficient in the field of targeted drug delivery. Over the course of three decades and through numerous studies on exosomes and their application as natural drug carriers in the nanoscale range their unique biological merits have been reported by the researchers.^10,11^ They have all the potentials and characteristics that could be expected from an effective drug carrier. These include proper drug loading capacity, high biocompatibility, ability to release the loaded drug, easy surface receptor modification, crossing tissue and physiological barriers of the body for their nanoparticle size.^1^

 The membrane nature of the exosomes allows them to efficiently interact with the cells and accurately and completely deliver their contents to the target cells. This is facilitated by the presence of receptors and protein structures (e.g., tetraspanins, and integrins) in the exosome membrane that cause proper drug interaction and delivery to the membrane surface and even into the cytosol of receptor cells.^12,13^ Exosomes are safe from phagocytosis by macrophages because of their natural membrane structure and high biocompatibility compared to synthetic drug carriers. As a result, they increase drug delivery to the target cells and augment half-life of the loaded drugs.^14^

 Different methods of loading drug into the exosomes are available, which follow two basic rules: loading the drug during the formation of exosomes and loading after the extraction of exosomes. Two of the best and most widely used methods of drug loading, related to the post-exosome isolation stage, are exosomal incubation and sonication.^14^

 In different skeletal system-related studies, including osteoporosis, critical size defects, bone fractures and rheumatoid arthritis, bone marrow mesenchymal stem cells (BMMSCs)-derived exosomes have been used, and the results showed their regeneration effects and effective reconstruction capabilities. More specifically, these exosomes regulate osteoblast activity and differentiation that improve osteoporosis and increase fracture healing.^1,15^

 Development of targeted therapies with high efficacy as well as low cytotoxicity for bone tissue is of high demand. Exosomes can be used as a promising drug delivery approach for osteoporosis because of their high biocompatibility, high drug loading capacity, efficient tissue targeting, and bone repair effects.^2^ In our current investigation we report the impact of two different methods of estradiol loading (incubation and sonication) in BMMSC derived exosomes.

## Materials and Methods

###  Chemicals and reagents

 Dulbecco’s Modified Eagle’s Medium (DMEM/F12), fetal bovine serum (FBS) and penicillin-streptomycin solution were purchased from GIBCO (Invitrogen Inc. Gibco BRL, USA). Trypsin/EDTA was provided from Life Technologies (Grand Island, NY, USA). 3-(4,5- dimethylthiazol-2-yl)-2,5-diphenyltetrazolium bromide (MTT) was purchased from Sigma-Aldrich (St. Louis, MO, USA). Bicinchoninic acid (BCA) protein assay kit was obtained of Santa Cruz Biotechnology (Texas, USA). HPLC-grade methanol (MeOH) (99.8%), acetonitrile (MeCN) (99.8%), and water (H2O) were purchased from Merk (Darmstadt, Germany). 17β-estradiol (99.6%) was provided from Sigma-Aldrich. 1 mg/mL of 17β-estradiol stock solution was prepared by dissolving an appropriate amount of the drug in methanol. All of the stock solutions were stored in the dark at -20 °C freezer. Experimental solutions were prepared freshly according to the study design by serial dilution.

###  Cell culture and characterization

 All methods, procedures, and experiments of working with animals were approved by the Animal Care and Committee of Ethics of the Tabriz University of Medical Sciences. The research team tried to use the minimum number of animals in the study, as much as possible. Three male New Zealand white rabbits (4-weeks old) were provided from the central animal house of TUOMS.

**Figure 1:**
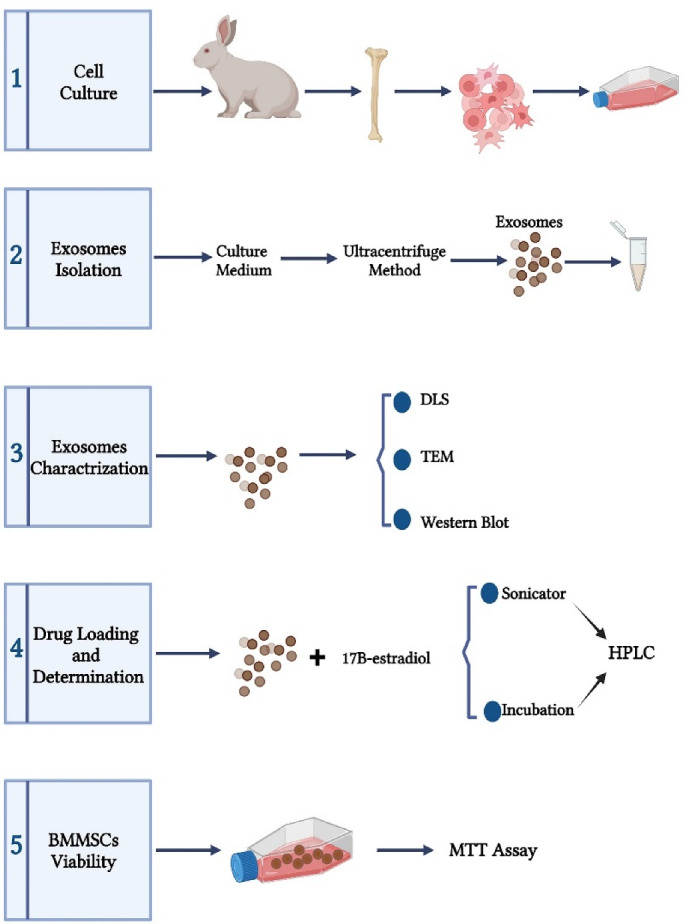
Schematic view of the study steps (The figure is drawn without considering the scale).

 As explained by Maniatopoulos et al,^16^ bone marrow was flashed out from the tibia and femur of male New Zealand white rabbits (4-weeks old) into a pellet and then, transferred to 15 mL Falcons tube and centrifuged to gain cell pellet and cell washing. Then, the cells were cultured in 75T flask with DMEM/F12 culture medium supplemented with 10% FBS and 1% penicillin/streptomycin at 37 °C under 5% CO_2_ humidified atmosphere. After 4 days, the culture medium was changed and changing medium happened every three days afterwards. After the cell density reached 80% to 90%, the cells were trypsinized, harvested and subcultured. BMMSCs in the third passage were used in experiments (Figure 1).

 After conducting three rounds of cell passages and in order to determine BMMSCs, cell morphology was examined with an inverted microscope (Olympus, Tokyo, Japan). Then, flow cytometry was utilized to determine cell surface markers. For this purpose, two positive (CD73 and CD90) (Abcam, Cambridge, UK) and two negative (CD45 and CD34) (Abcam, Cambridge, UK) factors were examined using FACSCalibur instrument (Becton Dickinson). Finally, data analysis was performed with FlowJo software.

###  Isolation of exosomes

 In the third cell passage and after reaching a confluency of 80% to 90%, the culture medium was removed and the BMMSCs were washed with *phosphate-buffered saline* (PBS) and then, the culture medium without FBS (starvation medium) was added to the flasks. BMMSCs were left at this condition for 48 hours. The medium was then collected and centrifuged (Olympus, Tokyo, Japan) at 300 × g for 20 min, followed by centrifugation of 2000 × g for 30 min and then 10 000 × g for 40 minutes to remove cell debris. Afterward, the supernatant was filtered with a 0.22-μm pore size sterile filter (Millipore, Billerica, MA). Then, the medium was ultracentrifuged (Beckman Optima TLX ultracentrifuge, Ramsey, US) at 110 000 × g for 75 minutes, and the supernatant was discarded and the exosomes were re-suspended in PBS, and again ultracentrifuged under the same conditions. Finally, by removing the supernatant, the exosomal pellet was re-suspended in 100 µL of PBS. The exosomal samples were stored at −80 °C until used (Figure 1). The protein content of the extracted exosomal samples was measured with a bicinchoninic acid protein assay (BCA) kit (Thermo Fisher Scientific Inc. Waltham, MA, USA) according to the manufacturer’s instruction.

###  Exosomes characterization 

####  Transmission electron microscopy

 The round morphology and 30-150 nm exosomes were examined under a transmission electron microscope (TEM, Zeiss, Germany). Formvar-coated 300-mesh copper grids were covered with a drop of the exosomal sample extracted from BMMSCs for 15 minutes at room temperature (RT). Then, the exosomal sample was negatively stained with 2% uranyl acetate and allowed to dry at RT. Finally, the grids were viewed with a Zeiss system with 100 kV (Figure 1).

####  Analysis of dynamic light scattering size 

 Three important indices of particle size, polydispersity index (PdI) and zeta potential of exosomal samples were examined by dynamic light scattering (DLS) method by a Nano ZS ZEN 3600 (Malvern, UK) device. The dispersion technology software (DTS) (Version. 6.01) from Malvern (Malvern Instruments, UK) was used for raw data analysis. Prior to each use of DLS method, the BMMSCs-exosome samples were sonicated at 4 °C for 10 minutes to eliminate the exosomal aggregates and completely separate exosomal particles. The exosomes were diluted and mixed with filtered PBS in a ratio of 1:5 and then, injected into the micro cuvette. The analysis of DTS demonstrated and showed the mentioned factors (Figure 1).

####  Western blot analysis

 Exosomes extracted from BMMSCs were examined by Western blot analysis for the expression of exosomal-specific protein factors (CD9, CD63 and Cytochrome c (Cytoc)). The primary and secondary antibodies were obtained from Santa Cruz (Santa Cruz Biotechnology, Texas, USA). The exosomes were lysed by lysis buffer for 45 minutes. The supernatant was centrifuged at 12 000 rpm for 4 minutes at 4 °C. The protein content was demonstrated by the BCA protein assay kit. Then, 20 mg of protein was loaded on 10% SDS-polyacrylamide gel and electrophoresed, and afterward, electro transferred to polyvinyl difluoride membranes. The membranes were blocked with 3% bovine serum albumin (BSA) in PBS at RT and then, with the primary antibodies (anti-CD9, anti-CD63 and anti-Cytoc) were incubated overnight at 4 °C. Next, the membranes were washed and incubated with secondary antibody (mouse anti-rabbit IgG-HRP) for 1 hour. The blots were depicted with a chemiluminescence detection system (Pierce ECL, Thermo Fisher Scientific), and finally the bands resulted from immunoreaction were imaged (Figure 1).

###  Loading drug in exosomes

 Initially, pure exosomes (100 μg) were mixed with 17β-estradiol (100 μg) in 100 μL of PBS to load 17β-estradiol into the exosomes. Two methods were applied for 17β-estradiol loading into the exosomes, including: 1) incubation at 37 °C and 2) sonication. In the first method, the exosome-drug mixture was incubated at 37 °C for 1 hour in shaking position. In the second method, initial mixture was sonicated using a Model UP50H Hielscher Ultrasonics (Germany, Teltow, Hielscher Ultrasonics, UP50H). The device was adjusted to 20% amplitude and 6 cycles of 30 s on/off for 3 min with a 2 minutes cooling period between each cycle. After sonication, the exosome-estradiol mixture was incubated at 37 °C for 1 hour to recover the exosomes membrane (Figure 1). The excess unloaded drug was separated from the exosome-drug mixture by size exclusion chromatography (SEC) using a Sephadex G-25 column device (Cytiva, Massachusetts, USA).

###  Determining drug loading in exosomes

 The measurement of 17β-estradiol loaded in BMMSCs-exosomes was performed using high performance liquid chromatography (HPLC) method. The stock solution was prepared by dissolving 17β-estradiol in methanol at the concentration of 1 mg/mL. It was serially diluted to final concentrations of 10, 50, 100, 500, 1000 µg/mL. Then, a calibration curve was established. Measuring 17β-estradiol loaded in BMMSCs-exosomes was performed based on Kim et al study.^17^ Briefly, the microtubes containing 17β-estradiol-loaded exosomes were placed on a heating block at 75 °C to evaporate the solution in the microtubes. An equal volume of acetonitrile was added, vortexed, and sonicated (to destroy the membrane structure of exosomes). The mixture and then centrifuged at 13 000 rpm for 10 min. Next, the supernatant was filtered through a corning cellulose 0.22 µm syringe filter and the filtered sample was transferred to vials. A 20 µL of sample solution was applied to the HPLC system (KNAUER, Germany). HPLC analysis was performed using a C18 column (C18, 250 mm x 4.6 mm, 5 µm, 100 Å, KNAUER, Germany) with a mobile phase consist of water (30%) and methanol (70%), flow rate of 1 mL/min at RT. Elution of analyte (17β-estradiol) was monitored using UV absorption 280 nm. The extent of17β-estradiol loading was expressed in ng of 17β-estradiol/100μg of exosomal protein as shown in Figure 1.

###  In-vitro assay of cell viability 

 The effects of exosomes, 17β-estradiol-loaded exosomes, as well as estradiol alone, on BMMSCs viability were assessed by MTT method. In summary, 10^4^ cells were placed in each well of 96-well microplate (Nunc, Roskilde, Denmark) and placed in an incubator for 24 hours (37 °C, 5% CO2 humidified atmosphere) to allow BMMSCs adhere to the wall. Wells with BMMSCs and normal culture medium were considered as controls. The assay was performed by adding 100 µL of MTT solution to each well followed by incubation for 2 hours. The culture medium and the remaining MTT solution were then removed from the wells and replaced with 100 µL of DMSO to dissolve the formed formazan crystals. Finally, the absorption was measured with a 570 nm wavelength by an ELISA plate reader (Roche Applied Science, Indianapolis, USA). The survival percentage of different groups was measured compared to that of the control group (Figure 1).

###  Statistical analysis

 For all the experiments, the data were presented as mean ± standard deviation (SD). Depending on the type of experiments, the utilized statistical tests may include t-test and one-way ANOVA with multiple comparisons performed using GraphPad Prism 9.0 (GraphPad software, San Diego, CA, USA). A minimum *P* value of 0.05 was selected as the significance level.

## Results

###  Phenotype characterization of BMMSCs

**Figure 2:**
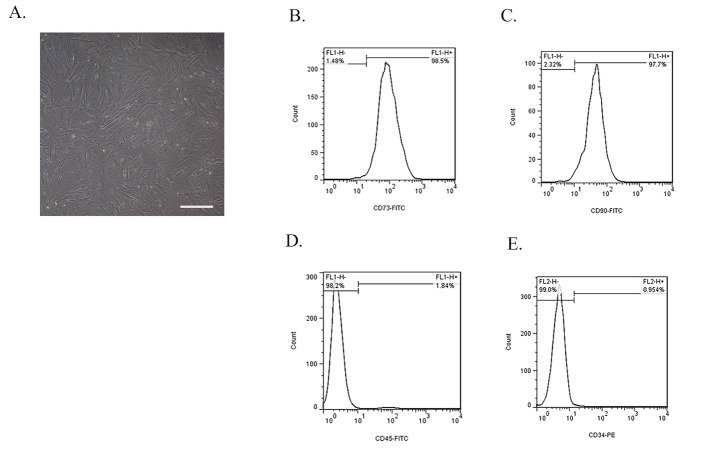
Phenotype characterization of BMMSCs. (A) Morphology of rabbit BMSCs exhibited a typical spindle-like shape under inverted microscope and also these cells were plastic adherent. Image scale bar: 100 μm. Flow cytometry analysis of surface markers of BMMSCs. These cells were positive for (B) CD73 and (C) CD90 and were negative for (D) CD34 and (E) CD45. The image and graphs are representative of the results of three independent experiments.

 BMMSCs were successfully extracted from the bone marrow of rabbits according to the above mentioned method. Cell confluence before each new passage was approximately 90%. BMMSCs showed a spindle-shaped morphology (Figure 2A), which was observed by inverted microscope (Olympus, Tokyo, Japan). Overall, the cells showed high density, spindle-shaped morphology, and wall-attached status. Flow cytometry showed that BMMSCs express high levels of CD73 (Figure 2B) and CD90 (Figure 2C) markers, while expression levels of CD45 (Figure 2D) and CD34 (Figure 2E) markers were very low.

###  Characterization of BMMSCs-derived exosomes

**Figure 3:**
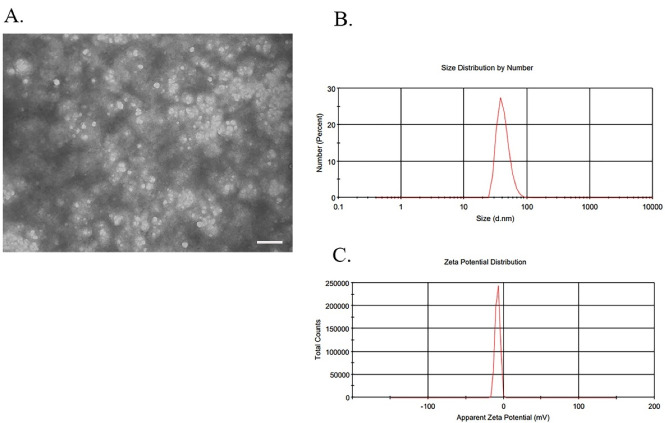
Characterization of BMMSCs-derived exosomes. Exosomes were extracted from rabbit BMMSCs by differential ultracentrifugation method. (A) The ultrastructure of Exosomes observed by TEM. Observations of BMMSC-exosomes represented the vesicular structures with a 30–150 nm in size. Image scale bar: 200 nm. (B) Size determining results by DLS of BMMSCs derived exosomes represented a heterogeneous population in the range of 30 to 150 nm. (C) Zeta potential was measured by DLS and the data showed a negative surface charge (about -10) for BMMSCs derived exosomes. The image and graphs are representative of the results of five independent experiments.

**Figure 4:**
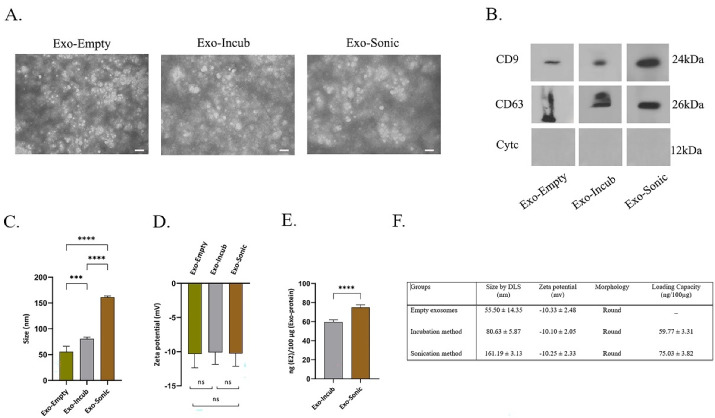
Characterization of BMMSC-exosomes loaded with 17β-estradiol. Exosomes were isolated from conditioned medium of BMMSCs, and loaded with 17β-estradiol by incubation and sonication methods. (A) Morphology of BMMSCs-exosomes (with and without drug loading) assessed by TEM. Image scale bar: 200 nm. (B) The expression of exosome-associated factors CD9, CD63 (positive markers) and cytochrome c (negative marker) was determined in BMMSC-exosomes (loaded and not loaded with drug) by western blot. Amount of protein factors (CD9 and CD63) was high, but there was no expression of cytochrome c protein in the exosomes of different groups. (C, D) The size and zeta potential of BMMSC-exosomes loaded with 17β-estradiol was determined by DLS. Data showed that the loading with drug increased the size of BMMSC-exosomes, but did not significantly changed their negative surface charge. (E) HPLC analysis was used to compare the amount of drug loaded in BMMSC-exosomes in sonication and incubation methods. The amount of drug loaded in sonication procedure was significantly higher compared to incubation. (F) Size, zeta potential, morphology and drug loading capacity have been shown in different study groups. The images and graphs are representative of the results of five independent experiments. (*** *P* ≤ 0.001; **** *P* ≤ 0.0001; ns, non-significant)

 The isolated exosomes from BMMSCs culture medium were examined for morphology (Figure 3A), size (Figure 3B), and zeta potential (Figure 3C). The size of the empty exosomes was 55.50 ± 14.35 nm in diameter, while it was 80.63 ± 5.87 and 161.19 ± 3.13 nm for drug-loaded exosomes prepared by the incubation and sonication methods, respectively (Figure 4C, 4F). TEM method showed that the shape of BMMSCs-exosomes was round, and did not change after loading the drug (Figure 4A). The zeta potential features of exosomes in different states (empty exosome and loaded exosome prepared by two different methods) are shown in Figure 4D and 4F. The negative zeta potential values (~-10 mV) for the prepared exosomes may be attributed to the anionic phosphatidylserine, which is abundant in cell and exosomal membranes. The results also showed that the zeta potential values for the exosomes in all conditions (exosomes alone and drug loaded exosomes) were almost the same indicating lack of significant change in the membrane structure and therefore, the surface membrane charge.

 Western blot analysis was applied to evaluate the surface markers (CD9, CD63 and Cytoc) of the extracted exosomes to evaluate possible changes in exosomal surface markers before and after drug loading (Figure 4B). The results showed high expression of surface protein markers (CD9 and CD63) in BMMSCs-derived exosomes. It was also observed that loading 17β-estradiol through sonication method did not significantly change the exosomal surface markers. The results of different conditions showed high expression of CD9 and CD63 (the positive markers) and lack of Cytoc expression (the negative marker) between the three groups.

###  Measurement of loaded 17β-estradiol in exosomes

 HPLC method was applied to measure the amount of encapsulated 17β-estradiol in the prepared exosomes. The amount of loaded drug was expressed as the amount of 17β-estradiol/100 µg of exosomal protein. It was found that drug loading efficiency by sonication and incubation methods were 75.03 ± 3.82 and 59.77 ± 3.31 ng/100 µg, respectively (Figure 4E, 4F).

###  The effect of exosomes on in-vitro viability of BMMSCs

**Figure 5:**
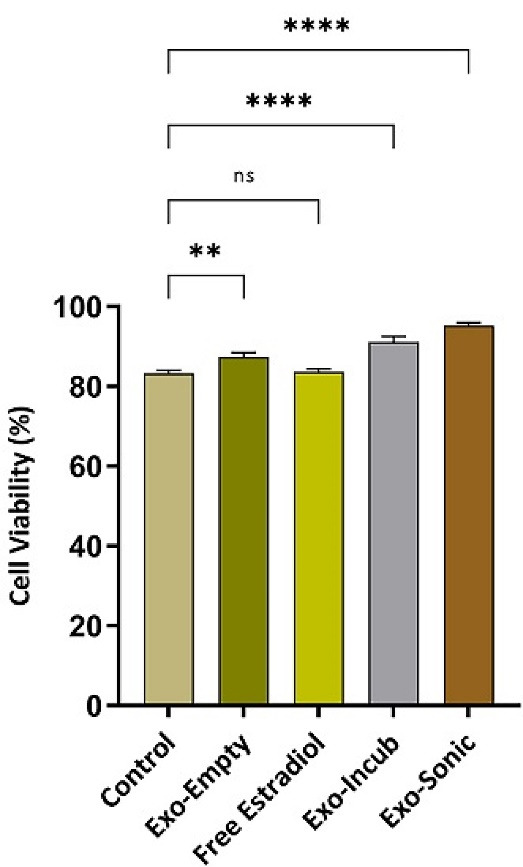
Viability of BMMSCs treated by different modes of drug and exosome. MTT assay was applied to measure viability of BMMSCs after (24 h) exposure to empty exosomes, free 17β-estradiol (100 µg) and exosomes loaded with 17β-estradiol (incubation and sonication procedures). The analyzed data represents the results of three independent experiments. (**P ≤ 0.01; ****P ≤ 0.0001; ns, non-significant)

 Since the survival of BMMSCs under osteoporosis conditions is significantly reduced, any possible treatment strategy should be able to increase the survival rate of these cells.^18^ Therefore, the effect of BMMSCs-derived exosomes, in different conditions with and without drug and also drug alone, on *in vitro* survival of BMMSCs was investigated by MTT method. The results of BMMSCs survival in different groups of study are shown in Figure 5. The data showed that BMMSCs survival rate after treatment with sonication-loaded exosomes was significantly (*P* ≤ 0.0001) increased compared to control group.

## Discussion

 Osteoporosis is a condition in which, due to the structural changes, bones become more fragile leading to frequent fractured bones caused by pressure and impacts. The severity of the condition increases progressively by age. Throughout the life, bones undergo structural changes in the form of removal of old tissues and formation of new replacement tissues. This process is performed by osteoclasts, as bone absorbing cells, and osteoblasts as bone forming cells in a very delicate and balanced manner. For various reasons, the normal homeostasis and metabolism of these cells and thus, the bone tissue is disturbed. Aging, menopause, long-term and high intake of glucocorticoids, and metabolic diseases are among the most important conditions that cause the loss of tissue balance and, as a result, higher bone resorption *versus* less bone formation.^19^

 In recent years, to treat osteoporosis, anti-bone resorption drugs such as: estrogens, bisphosphonates, calcitonin and others have been prescribed. These agents applied for both prevention and treatment of osteoporosis. They reduce bone resorption by osteoclasts and increase bone production by osteoblasts. These effects in long term increase bone mass and strength and reduce the occurrence of fractures.^19^ Drug treatments in this manner and regarding inflammatory diseases, are based on the utilization of drugs to target the desired tissues. However, the effect left by these drugs in these situations will be on both inflamed and healthy tissues. Inducing a therapeutic effect on the target inflammatory tissues in most cases requires prescribing and using high doses of drugs, which can also cause unwanted effects on healthy tissues.^20^

 Estrogen is a key factor in the size and shape of the skeleton during growth. It also plays an effective role in the establishment of skeletal homeostasis during adulthood.^21^ Estrogen causes desired changes by affecting its receptors in target cells. Estrogen receptors (ERs) are the members of the superfamily of ligand-regulated nuclear transcription factors. Two types of estrogen receptors including ER-α and ER-β are known, and they exist in bone mesenchymal stem cells (MSCs), osteoblasts, osteoclasts and also in precursor cells of both types.^22^ The normal level of estrogen in the body is vital and consuming large amounts of estradiol (for the treatment of osteoporosis) in the long term can cause serious complications in other tissues. Ischemic arterial disease, breast cancer, endometrial cancer, cervical cancer, prostate cancer, laryngeal papillomatosis, abnormalities in the lower urinary tract and accessory sex glands are among these complications.^9,23-25^

 In recent years, investigations on drug delivery systems in the field of nanotechnology in relation to different disorders such as cancers have suggested promising and feasible results.^17^ The most key studied nanocarriers in the field of nanotechnology come from two origins, synthetic and non-synthetic.^26^ It has been observed that exosomes, as the parts of non-synthetic (natural) carriers, have unique abilities in loading and targeting drugs. Specifically, they have a particular ability to interact and release drugs into the target cell/tissue. According to the studies, exosomes possess certain properties based on the origin tissue, which should be considered depending on the type of target cell/tissue being delivered.^17^

 Exosomes have the merits of both types of drug treatment systems, i.e. synthetic drug carriers and cell-mediated drug delivery and at the same time, they don’t have the demerits of both. Compared to synthetic carriers, exosomes prevent rapid clearance, have high biocompatibility, do not have their toxic effects, and do not cause immune responses. When compared to the cell-mediated drug delivery system, they have more accurate drug transfer and homing, and do not have its clinical complications. All these features highly suggest the use and development of exosomes as targeted drug carriers both in researches and clinic.^17^

 In recent years, MSCs-extracted exosomes have been utilized in many studies in the field of regenerative medicine and drug therapy with promising results. The use of BMMSCs-exosomes in bone research, especially osteoporosis, has been widely used with highly positive results. The same histological and anatomical origin of BMMSCs and their exosomes, and as a result, similarity of structural, factors and processes involved in osteogenesis between them (BMMSCs-Exosome and targeted bone tissue), could be an effective factor in achieving these results.^2,18,27-32^ also in the current study, exosomes isolated from BMMSCs.

 Ultracentrifugation is a method applied to extract exosomes. It is considered as “gold standard”, which has been used in more than 50% of studies on exosomes isolation.^33^ It enjoys advantages such as: high yield, low cost, and scalability.^33^ Ultracentrifugation has been used in different studies to extract exosomes from BMMSCs and their application in osteoporosis studies.^2,18,27,28^ In the present study, similar to the studies, the method was utilized for the same objective.

 Working with exosomes requires high precision. One of the things that should always be considered is that loading desired drugs in exosomes should be in a way that it does not leave significant structural and especially membrane changes in exosomes.^17^ In this research, two types of drug loading methods were used in exosomes, including: incubation at 37 °C and sonication. Sonication caused more (*P* ≤ 0.0001) drug loading compared to incubation at 37 °C. 17β-estradiol is a compound with a strong hydrophobic property. Like other studied hydrophobic compounds such as PTX,^17,26,34^ it incorporates into the inner hydrophobic region of the bilayer membrane of exosomes during drug loading and is placed there. By performing sonication, the uniformity and stiffness of the lipid membrane of exosomes is reduced, and finally, 17β-estradiol incorporates into the membrane of exosomes, and in this way, it causes more drug loading than incubation. The same results were observed in other studies by performing sonication to load hydrophobic drugs such as PTX into exosomes.^17,26,34^

 In this study, TEM and DLS methods were used to specify the morphology, size, and zeta potential. TEM showed the round morphology and approximate size of exosomes to be in the range of 30 to 150 nm. This size range was also confirmed by the DLS method (Figure 4C, 4F). The round shape was constant in all exosomes (pure exosome or drug-loaded exosome). The size and shape results in this study were completely consistent with the results reported by other researches.^17,20,26,34,35^

 According to the results in Figure 4C, the size of the exosomes loaded with 17β-estradiol was greater (*P* ≤ 0.0001) by the sonication than the incubation. This can be due to membrane modification of exosomes resulting from sonication. The same results and discussions have been achieved in other researches.^17,26,34,36^ In connection with the zeta potential obtained by DLS, as stated, the significant changes of zeta potential in BMMSCs-exosomes were not observed with drug loading indicating no change (non-significant) in the negative charge of exosomes by 17β-estradiol loading. Loading drugs in pharmaceutical nanocarriers such as exosomes with different cellular origins, niosomes, etc., are effective on their size because of the surface adsorption of these particles.^34^ However, in the current study, no significant change was observed after 17β-estradiol loading in the negative charge of BMMSCs-exosomes.

 Many studies have examined the effects of 17β-estradiol. The results indicated the protective antioxidant effect of estradiol on MSCs, the stimulation of bone differentiation of MSCs, increased proliferation and viability, and decreased apoptosis of MSCs.^8,22,37-39^ The survival effect of exosomes loaded with 17β-estradiol was higher than that of control group and 17β-estradiol alone (*P* ≤ 0.0001). It can be due to the way exosomes interact with BMMSCs. This exosome-cell interaction occurs by merging the exosomes’ membrane with cells and discharging exosomes’ content into the cell, or complete endocytosis of exosomes and their contents by cells.^17,26,34,36^ Compared to other studies, similar results were achieved in which the effect of survival and cytotoxicity of the drug loaded with exosome was greater than the drug alone.^17,34,35^ In fact, as previously stated, the key element in the effect of high survival of exosomes on BMMSCs is the direct communication among them and the discharge of exosome contents into the target cell or endocytosis of the exosomes into the target cell.^17,34,35^

 In osteoporosis, there is a high combination of pro-inflammatory factors (IL-1, TNF-α, IL-6, IL-11, IL-15, and IL-17) and active osteoclastogenesis, which results in an inflammatory environment with low pH (acidic microenvironment) in the bone tissue.^4,40-42^ Parolini et al reported that the homing and fusion of exosomes to cells and tissues with an inflammatory (acidic) state occurs more frequently. In other words, inflamed areas such as: bone tissues in osteoporosis, tumors and other cases, for exosomes, have more transport and functional preference than healthy tissues and around the inflammatory area.^17,34,35,43^ Also, in addition to the mentioned cases, unlike synthetic nano drug carriers, exosomes have adhesion molecules and surface proteins (tetraspanins, integrins, immunoglobulins, proteoglycans, lectins and etc.), which cause higher general absorption and interaction of exosomes with their target cells.^17,34,36^

 Suggested by the results of the present study and other researches, exosomes and their drug loading activity could be significantly important for drug targeting in several ways. First, as mentioned, exosomes with different cell sources can have different reparative effects on the target tissue. For example, BMMSCs-exosomes have shown their excellent osteogenesis effect in different studies, apart from their ability to transport drugs; exosomes have restorative effects on their own.^18,27,28^ Second, 17β-estradiol carried by exosomes has survival, proliferative, and anti-apoptotic effects on MSCs, which can be very effective in the process of osteoporosis.^20,37-39^ Third, unlike synthetic nano carriers, exosomes do not have immune stimulation, toxicity, etc., making them a highly suitable option than synthetic carriers.^17,20^ Fourth, in osteoporosis, exosomes have a tendency to act and transfer to inflammatory areas such as bone tissue, which causes very precise drug targeting.^17,34,35^ Fifth, according to the type of therapeutic approach in different diseases, for example, the need to use several drugs to act in several molecular pathways (simultaneously), and in one type of cell in diseases, there is this ability for exosomes that the more number than one drug be loaded into them.^20^

 Despite all, more research should be done *in vitro* and especially *in vivo*, to know the extent and function of BMMSCs-derived exosomes loaded with 17β-estradiol on osteoporosis. The present study is a basic and preliminary research in the field of drug targeting systems for 17β-estradiol with a nano size range, which may have the potential to be used as a precise drug delivery method in the treatment of various diseases, including osteoporosis.

## Conclusion

 In summary, 17β-estradiol-loaded BMMSCs-derived exosomes can be regarded as a potentially effective therapy for patient with osteoporosis. These estradiol-containing nanoparticles can overcome many challenges associated with other drug delivery systems such as synthetic carriers and provide high biocompatibility, proper drug delivery and cell targeting, as well as drug protection. In this study, we have shown the significantly increased survival rate of BMMSCs treated with 17β-estradiol-loaded exosomes in comparison with the control group (almost 10% more survival rate). However, additional *in vitro* and *in vivo* studies are required before advancing the proposed approach into human clinical trials.

## Acknowledgments

 The authors are very grateful and appreciative to the staff of the Physical Medicine and Rehabilitation Research Center, Aging Research Institute, Tabriz University of Medical Sciences for their help and support.

## Competing Interests

 The authors declare that they have no conflict of interest.

## Ethical Approval

 This study was approved by the Animal Care and Committee of Ethics of the Tabriz University of Medical Sciences (TUOMS) (ethical code: IR.TBZMED.REC.1398.039).
